# Clinical Profile of Patients with Infertility Presenting to Tertiary Care Center: An Observational Study

**DOI:** 10.31729/jnma.8925

**Published:** 2025-03-31

**Authors:** Jyotshna Sharma, Dipty Shrestha, Durga Thapa, Sirjana Rawal, Rijan Kafle

**Affiliations:** 1 Department of Obstetri cs and Gynecology, Kathmandu Medical College and Teaching Hospital, Sinamangal, Kathmandu, Nepal; 2 Kathmandu University School of Medical Sciences, Dhulikhel, Kavre, Nepal; 3 BP Koirala Institute of Health Sciences, Dharan, Sunsari, Nepal

**Keywords:** *hormonal analysis*, *hysterosalpingography*, *infertility*, *semen analysis*

## Abstract

**Introduction:**

Infertility, defined as the inability to conceive after a year of regular unprotected sexual activity, is a significant global health concern. It affects couples across the world, with prevalence rates varying between 3.5-16.7% in developed countries and 6.9-9.3% in developing nations. This study aims to explore the sociodemographic profiles and contributing factors in both men and women among infertility patients at a tertiary care center.

**Methods:**

An observational cross-section study was conducted at a tertiary care center and participants were between 18 and 42 years old with infertility for one year or longer, excluding those who had received treatment or were pregnant. The data was collected from December 2023 to May 2024. Ethical approval and informed consent were obtained. Data were collected using a structure proforma through in-person interviews or telephone calls and analyzed using Statistical Package for the Social Sciences with descriptive statistics.

**Results:**

Among 170 couples with infertility, primary infertility was observed in 124 (72.94%) and secondary infertility in 46 (27.06%). The median duration of marriage was 5 years (IQR: 4-7). Among women, 37 (21.76%) had tubal blockages, 16 (9.41%) had polycystic ovarian disease, and 6 (3.53%) had fibroids. Among men, 58 (34.12%) had semen abnormalities, with oligospermia being the most common in 25 (14.71%). Hormonal analysis revealed that 68 (40%) of women had elevated anti-Müllerian hormone levels, frequently linked to polycystic ovarian syndrome.

**Conclusions:**

These findings show that infertility is rarely due to a single cause. It commonly involves both partners and requires a comprehensive approach to diagnosis and treatment.

## INTRODUCTION

Infertility refers to not achieving pregnancy after one year of regular sexual life without using contraception techniques.^1^ Infertility is one of the major healthcare problems in all societies worldwide. The average prevalence of infertility in developed countries is 3.516.7% and in developing countries is 6.9-9.3%.^2^ In South Asia, infertility is compounded by limited access to reproductive services and cultural stigmas, as highlighted by studies in Dhaka^2^. Similarly, disparities in infertility care provision exist in Europe, with uneven access to assisted reproductive technologies.^3^

In Nepal, socio-economic and cultural factors significantly influence fertility patterns, yet research on the clinical profile of infertility remains scarce.^4^

While studies have explored the psychosocial impact of infertility, there is a lack of data on the clinical characteristics and etiologies among patients in tertiary care settings.^5^ This study aims to bridge this gap by examining the clinical profile of infertility patients at a tertiary care center in Nepal.

## METHODS

This observational cross-sectional study was conducted at Kathmandu Medical College and Teaching Hospital (KMCTH), a tertiary care center in Kathmandu, Nepal. The data was collected from December 2023 to May 2024. The study aimed to explore the clinical profile of infertility among individuals aged 18-42 years who were unable to conceive after ≥1 year of regular, unprotected intercourse. The age range was selected to focus on the reproductive age group most likely to seek infertility care. Couples presenting with primary infertility (no prior conception) or secondary infertility (prior conception but inability to conceive again) during the study period were included. Individuals who had undergone infertility treatment or were currently pregnant were excluded to focus on untreated cases.

Before data collection, ethical approval was obtained from KMCTH's Institutional Review Committee (reference number 03112023/01). The ethical approval process included a thorough review of the study protocol, ensuring compliance with international ethical standards. Informed consent was carefully obtained from all participants, who were fully informed about the study's purpose, procedures, and risks. The consent process involved a detailed explanation of the study's objectives, potential benefits, and risks, as well as the voluntary nature of participation. Participants were assured of confidentiality, anonymity, and the right to withdraw at any time without negative consequences, ensuring trust and accurate reporting. To maintain confidentiality, all data were stored securely, with access limited to the research team.

Data were collected using a structured proforma through in-person interviews or via phone by the researchers. The proforma was developed based on a review of existing literature and expert consultation. The proforma covered demographic, medical, and lifestyle factors. Infertility diagnoses were based on clinical history, laboratory tests (e.g., hormonal assays), and imaging findings (e.g., hysterosalpingography). Semen analysis followed WHO 2010 guidelines, with abnormalities defined as sperm concentration <15 million/mL, motility <40%, or morphology <4% normal forms.^6^

Data were analyzed using SPSS Statistics for Windows. Descriptive statistics summarized categorical variables as frequencies and percentages, while continuous variables were reported as medians and interquartile ranges (IQR) due to non-normal distribution.

## RESULTS

The study included 170 couples with infertility, with a median age of 29 years (IQR: 27-32) for females and 33 years (IQR: 30-36) for males. The majority of females, 74 (43.53%), were aged 26-30 years, while most males, 65 (38.24%), were aged 31-35 years. The median duration of marriage among the couples was 5 years (IQR: 4-7), (Table 1).

**Table 1:** Sociodemographic profile of the participants (n=461).

Age Group (Years)	Female n (%)	Male n (%)
≤ 25	23 (13.53)	2 (1.18)
26 - 30	74 (43.53)	54 (31.76)
31 - 35	59 (34.71)	65 (38.24)
36 - 40	12 (7.06)	47 (27.65)
≥ 41	2 (1.18)	2 (1.18)
Total	170 (100)	170 (100)

Primary type of infertility was seen in 124 (72.94%) couples. Tubal blockage was seen in 37 (21.76%) females. Semen analysis was normal in 112 (65.88%) and abnormal in 58 (34.12%). Ultrasonogrpahy findings was normal in 137 (80.59%) followed by PCOD 16 (9.41%) (Table 2).

**Table 2:** Clinical Profile of infertility among study participants (n=170).

Categories	n (%)
**Infertility Type**	
Primary	124 (72.94)
Secondary	46 (27.06)
**Hysterosalpingography Findings**	
Normal	133 (78.24)
Right Tubal Block	11 (6.47)
Left Tubal Block	13 (7.65)
Bilateral Tubal Block	13 (7.65)
**Semen Analysis**	
Normal	112 (65.88)
Oligospermia	25 (14.71)
Azoospermia	4 (2.35)
Asthenoteratospermia	20 (11.76)
Teratospermia	9 (5.29)
**Ultrasonography Findings**	
Normal	137 (80.59)
Polycystic Ovarian Disease (PCOD)	16 (9.41)
Fibroid	6 (3.53)
Endometriosis	4 (2.35)
Adenomyosis	6 (3.53)
Bicornuate Uterus	1 (0.59)

Out of total male, 25 (14.71%) had oligospermia, 4 (2.35%) had azoospermia, 20 (11.76%) had asthenoteratospermia, and 9 (5.29%) had teratospermia (Table 3).

Follicle-stimulating hormone (FSH) and luteinizing hormone (LH) median values were 4.72 IU/L (IQR: 3.86-6.20) and 5.27IU/L (IQR: 3.40-8.47), respectively. Thyroid-stimulating hormone (TSH) and anti-Müllerian hormone (AMH) median levels were 2.50mU/L (IQR: 1.68-4.92) and 2.70 ng/ml (IQR: 1.504.90), respectively. The distribution of Anti-Müllerian Hormone (AMH) was high among 68 (40%), normal among 77 (45.29%) and low among 25 (14.71%), (Table 3). Median prolactin level was 13.74 (IQR: 10.11-16.48).

**Table 3:** Semen analysis of male participants according to age group(n = 170).

Age Group	Normal n (%)	Oligospermia n (%)	Azoospermia n (%)	Asthenoteratospermia n (%)	Teratospermia n (%)	Total n (%)
≤25	2 (1.18)	-	-	-	-	2 (1.18)
26 - 30	32 (18.82)	9 (5.29)	1 (0.59)	6 (3.53)	6 (3.53)	54 (31.76)
31 - 35	42 (24.71)	10 (5.88)	3 (1.76)	7 (4.12)	3 (1.76)	65 (38.24)
36 - 40	35 (20.59)	6 (3.53)	-	6 (3.53)	-	47 (27.65)
≥ 41	1 (0.59)	-	-	1 (0.59)	-	2 (1.18)
Total	112 (65.88)	25 (14.71)	4 (2.35)	20 (11.76)	9 (5.29)	170 (100)

## DISCUSSION

In our study, the propotion of the primary infertility was greater than that of the secondary infertility affecting nearly three out of every four couples (72.94%). This aligns with studies such as Pradhan et al. (62.50% primary infertility) but contrasts with findings from Nigeria, where secondary infertility was more common due to a high burden of genital tract infections.^9,10^ This discrepancy might have been resulted from influence of regional factors, such as healthcare access, environmental conditions, and cultural practices, on infertility patterns.

One of the most common findings in our study was polycystic ovarian disorder (PCOD), detected in 9.41% of women. This finding of ovulatory dysfunction resulting in infertility is consistent with global trends.^8^ However, tubal blockage emerged as the leading cause of female infertility in our cohort (21.76%), similar to findings by Deshpande et al. (20%).^13^ This contrasts with studies by Carson et al., where ovulatory disorders (25%) were the primary cause, suggesting regional variations in infertility etiologies.^14^ The cause of high proportion of tubal factors as a reason for infertility in our study and similar region remains yet to be evaluated.

There were 34.12% of men exhibiting abnormal semen parameters. Azoospermia, the absence of sperm in the ejaculate, was identified in 2.35% of cases, while asthenozoospermia and oligospermia were also prevalent. These findings are comparable to studies by Pradhan et al. and Panti et al., though the prevalence of azoospermia was lower than in study from Nigeria 12.6%.^9,10,15^ This variability may be accounted to differences in diagnostic criteria, lifestyle factors, or genetic predispositions across populations.

The demographic profile of our study population revealed that the majority of women (43.53%) were aged 26-30 years, while most men (38.24%) were aged 31-35 years. This aligns with findings from other studies, reflecting the typical age range of couples seeking fertility evaluation.^9^^^2^ The median duration of marriage was 5 years, indicating that many couples had been attempting conception for several years before seeking medical intervention. This delay may be influenced by cultural factors, as children are highly valued in Nepalese society, symbolizing social and economic well-being.^4^ However, the rising prevalence of infertility (9.1% as per Regmi et al.) suggests that changing lifestyles, environmental pollution, and delayed marriages may be contributing factors.^5^

Hormonal analysis provided further insights into ovulatory dysfunction and ovarian reserve. The median levels of luteinizing hormone (LH: 5.27), follicle-stimulating hormone (FSH: 4.72), anti-Müllerian hormone (AMH: 2.70), and thyroid-stimulating hormone (TSH: 2.50) were consistent with findings from Pradhan et al., indicating that hormonal imbalances are a significant contributor to infertility in this population.^9^ Elevated prolactin levels (median: 13.74) were also observed, which can disrupt ovulation and further complicate fertility. Despite these findings, our study has limitations. As a single-center study, the results may not be generalizable to other regions of Nepal or countries with different healthcare systems and cultural contexts. Additionally, while we identified common causes of infertility, we did not explore potential underlying factors such as genetic predispositions, environmental exposures, or lifestyle behaviors. Future research should aim to address these gaps by conducting multi-center studies with larger sample sizes and incorporating advanced diagnostic tools.

## CONCLUSIONS

This study highlights primary infertility as the predominant type among couples seeking care, with tubal blockage and PCOD being common causes in females. Male factor infertility was observed in over one-third of cases, with oligospermia and asthenoteratospermia being frequent findings.

The demographic and hormonal profiles align with regional trends, emphasizing the multifactorial nature of infertility in Nepal.
